# Impact of Sled-Integrated Resisted Sprint Training on Sprint and Vertical Jump Performance in Young U-14 Male Football Players

**DOI:** 10.3390/jfmk9040256

**Published:** 2024-12-05

**Authors:** Manuel Amore, Diego Minciacchi, Giulia Panconi, Sara Guarducci, Riccardo Bravi, Vincenzo Sorgente

**Affiliations:** 1 Kinesiology and Motor Control (Ki.Mo.Co.) Laboratory, Department of Experimental and Clinical Medicine, Physiological Sciences Section, University of Florence, 50134 Florence, Italy; manuel.amore@edu.unifi.it (M.A.); giulia.panconi@unifi.it (G.P.); riccardo.bravi@unifi.it (R.B.); 2 Department of Information Engineering, University of Florence, 50121 Florence, Italy; sara.guarducci@unifi.it

**Keywords:** sprint training, jump capabilities, combined training, resistance training, power, explosive performance, countermovement jump, strength training, youth, boys

## Abstract

**Background/Objectives**: The aim of this study was to investigate the effects of a six-week integrated resisted sprint training (IRST) program on sprint performance and vertical jump height in a sample of U-14 male football players. This study also explored the potential benefits of incorporating variable resistive loads during pre-peak height velocity (pre-PHV) developmental stages, a period often overlooked in the training of young athletes. The IRST program alternated between heavy and light resistive sled loads to enhance sprint and jump capabilities, which are critical components of athletic performance in football. **Methods**: Nineteen healthy male football players (age: 13 ± 0.63 years) were divided into an experimental group (E, *n* = 10) and a control group (C, *n* = 9). The experimental group followed the IRST protocol, involving sled sprints with varying resistive loads (10–115% of the body mass) over specific distances, while the control group engaged in traditional unresisted sprint training. The sprint performance was assessed using 30 m sprint times, and the vertical jump height was measured using countermovement jump (CMJ) data collected via a force platform. Anthropometric measures and peak height velocity (aPHV) estimates were also recorded pre- and post-intervention. **Results**: The experimental group demonstrated significant improvements in 30 m sprint times (mean difference: −0.29 s; *p* < 0.01). Additionally, CMJ data revealed a positive trend in the take-off velocity and maximum concentric power, with an increase in jump height (mean difference: +0.44 cm). These results suggest enhanced sprint and explosive power capabilities following the IRST intervention. **Conclusions**: The findings suggest that the IRST program is an effective training method for enhancing sprint performance and maintaining jump capabilities in young football players. This approach highlights the importance of integrating variable resistance training in pre-PHV athletes to promote athletic development while ensuring safety and effectiveness.

## 1. Introduction

Sprinting is defined as running over a short distance at the top-most speed of the body [1]. In particular, sprinting is defined as such when it is performed at a velocity of ≥7 m/s [2]. It is worth noting that the sprint is one of the most crucial gestures in football [3]. The high frequency and critical nature of sprints in football have led to extensive research into techniques and/or methodologies to enhance sprinting performance [4,5,6]. Specifically, several researchers have focused their studies on improving the sprint gesture of younger footballers, since acquiring and mastering proper technique during the early years of sporting activity can significantly reduce injuries and optimize running mechanics and performance overtime [7]. Within this context, sled-resisted sprint training (sled-RST) has emerged as a promising method for enhancing both specific strength and maximum sprint velocity [8]. In this regard, research has primarily concentrated on lower training loads for sled-RST. In fact, sled-RST protocols are typically performed over longer sprinting distances (>20 m) and utilize low resistances, not exceeding 20% circa of an athlete’s body mass [9,10,11]. This approach has been shown to reduce sprint times over distances of 20 and 30 m [12,13,14], enhance maximum sprint speed, and improve vertical jump performance [15,16,17].

On the other hand, heavy-load sled-RST protocols have also shown some results in ameliorating overall sprinting performance. Physiologically, high-load training, typically defined as lifting loads near one’s one-repetition maximum (1RM), is known to yield significant strength and power gains compared to lower resistances [18,19,20]. In the context of sled-RST, heavier loads (greater than 20% of body mass) have been shown to positively impact acceleration and optimize running biomechanics [9,11,13]. Additionally, sled-RST with heavy loads has been shown to influence total strength, explosive power, and muscle endurance [20]. Moreover, sled training with heavier loads positively impacts acceleration and contributes to the optimization of running biomechanics [9,21,22]. In this context, Petrakos et al. (2016) conducted a systematic review, highlighting the potential benefits of resisted sled sprint training (RSS) over traditional unresisted sprint (URS) training. Their findings suggest that heavier sled loads can significantly improve acceleration phases, although the advantages of RSS training over URS training are still debated, necessitating further research into optimal load prescriptions for different athletic populations [23].

This would suggest that an integrated approach, alternating and combining different loads and distances, could be the most effective strategy for enhancing the sprint performance of younger footballers. It is worth noting that the aforementioned approach would be in line with the current state of the art regarding the optimal periodization of fundamental bio-motor abilities such as strength, power, and endurance [23].

However, there is a lack of data on the effects of sled-RST with high loads (e.g., greater than 60% of body mass) in young football players aged 13–14, despite the absence of evidence indicating that submaximal or even maximal training loads are a direct cause of stunting. In fact, the American Academy of Pediatrics recommends strength training for kids 8 years old and up as a safe way to build strength [24]. A final issue to be considered is the age of subjects. Existing recommendations for strength training consider starting 12–18 months after the estimated age peak of height velocity (aPHV) [25,26] while no data on sled-RST with loads greater than 60% of body mass are available for young football players aged 13 and 14. No arguments are available regarding possible variations in the age of PHV estimation.

The key distinction between our study and the existing literature is that we did not employ a single type of load during the six-week training intervention with the athletes. Additionally, the distances covered while towing the sled varied depending on the different resistive loads used. To train at maximum speed, we used resistive loads not exceeding 20% of each athlete’s body mass, with the distances covered exceeding 20 m. To improve the power production, we employed resistive loads that were as close as possible to the peak power output over distances of approximately 15 m. To develop specific strength, we used loads greater than 80%, which were towed for a maximum distance of 10 m.

Given these considerations, the purpose of this study is to investigate the impact of sled-integrated RST on both sprint and vertical jump performance in a population of U-14 male football players. We hypothesize that sled-RST performed with higher loads will significantly improve both sprint performance and vertical jump capacity in this age group. Ultimately, this study aims to empower scholars, trainers, and practitioners in the realm of football with novel insights into effective methods for enhancing the performance and motor capabilities of their younger athletes.

## 2. Materials and Methods

### 2.1. Participants

A total of 19 young males (age 13 ± 0.63) participated in the study. None of these subjects had physical, cognitive, or motor disabilities. Before the study began, written informed consent was obtained from the parents/legal guardians of the participants. Additionally, the minimum number of participants required for the experiments was determined by an a priori power analysis using G*Power (version number 3.1.9.6), estimating a sample size of 18 subjects (f = 0.5, alpha at 0.05, with 75% power for ANOVA repeated measures within factors); therefore, we conservatively recruited forty young men to participate in this study. The study included an entire team of 19 U-14 male football players to reflect real-world training contexts and to prevent selection bias. Using the entire team aligns with the practical considerations of maintaining an intact training environment, which we deemed essential for the applicability of the findings. The anthropometric characteristics are reported in the Section 3 (Table 1) since it includes the pre- and post-intervention status.

### 2.2. Procedures

Prior to familiarization, subjects underwent a set of non-invasive measurements of their weight (SECA mechanical column scale MOD. 756, Hamburg, Germany), their height and their sitting height (SECA 206 Wall-Mounted Stadiometer, Hamburg, Germany) to estimate the maturation status and the estimate of aPHV. The aPHV was calculated using Mirwald and colleagues’ equation [27].

The countermovement jump (CMJ) was evaluated according to [28]. The CMJ assessment began with the participants standing with their hands on their hips. The participants were instructed to perform a CMJ by simultaneously flexing the hips and knees to a self-selected depth, then explosively jumping as high as possible, with the hands remaining on the hips throughout the execution period, and landing in the same position on the mat, with initial contact on the toes. Measurements related to the jump were obtained by the INFINI-T force platform (sensitive area: 60 × 40 cm, capacity ± 8000 N, sensitivity/resolution: 16 bits over the selected range, sampling frequency: 1000 Hz, BTS Bioengineering Corp., Italy; https://www.ultramedsrl.it/project/infini-t/, accessed on 11 June 2024) and by the inertial measurement unit (IMU, BTS G-Sensor 2, BTS Bioengineering Corp., Garbagnate Milanese, Italy; https://www.ultramedsrl.it/project/g-walk/, accessed on 11 June 2024). All participants performed 2 jumps interspersed with 2 min of rest. The largest jump was used for data analysis. The CMJ was evaluated at the beginning and end of the study, as well as the measurement of height and weight.

Additionally, for each subject, the force–velocity (F-V) profile was determined through a 30 m sprint test. A High Dynamic Range (HDR) video recording system with Dolby Vision up to 4K at 60 fps on an iPhone 13 Pro Max was used. The collected data were obtained by conducting the sprint test at the beginning of the study and at the end of the study.

**Figure 1:**
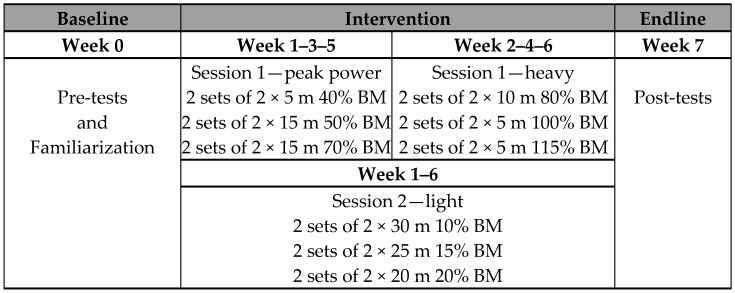
Experimental design, pre and post-test.

The participants were divided into two groups: 10 were allocated to the experimental group (E) and underwent sled-RST with loads from 10% to 115% of their BM; and 9 were allocated to the control group (C) and underwent an unresisted sprint training (Figure 1). Group assignment was performed using the Random Allocation Software (https://random-allocation-software.software.informer.com, accessed on 11 June 2024, version number 1.0).

Preceding the training period, group E subjects were exposed to a two-week familiarization program (Figure 1). During the first week, subjects completed 2 sets of 2 unresisted sprints, 2 sets of 2 sleds [Lacertosus, model: 0805698475372] sprints (0% of BM), and 2 sets of 2 sled sprints (20% of BM). In the second week, subjects completed 2 sets of 2 sled sprints (20% of BM), 2 sets of 2 sled sprints (40% of BM), and 2 sets of 2 sled sprints (80% of BM). Sprints were performed over 20 m. For the estimate of the external load, the coefficient of friction was measured. The coefficient of friction (µk) in the sled sprint was calculated using the following equation: µk = Ff/Fn, where Ff represents the horizontal tensile force (N) and Fn is the normal force [29].

Post-intervention, all the E and C participants were subjected to the same tests performed before the familiarization.

Participants’ heights were measured at the beginning and end of the study using a stadiometer [SECA 206 Wall-Mounted Stadiometer, Hamburg, Germany] and were used to calculate the PHV using predictive equations.

#### 2.2.1. E Group

Two weekly sessions were incorporated into the players’ traditional in-season program. No extra sessions were added to the three weekly routine training days. The intervention, lasting a period of 6 weeks, consisted of two weekly sprint-focused workouts (for a total of 12 sessions) which were added to the normal training program. The two intervention sessions were separated by 48 h of complete rest.

The experimental design was based on the training pyramid system [30]. The first weekly session included the use of heavy sled towing, alternating with a week with loads from 40% to 70% of BM (heavy session, Figure 1) and the next week with loads from 80% to 115% of BM (alternate heavy session, Figure 1). Participants completed 3 sets each, consisting of 2 sled sprints over distances depending on the load they towed in the session: Figure 1). Recovery between each repetition was assessed at 2 min, while recovery between sets was 3 min. On the second training day of the weekly protocol, loads from 10% and 20% of BM (light session, Figure 1) were used. Participants completed 3 sets of 2 sprints with the sled over 20 m, 25 m, and 30 m. Here, the recovery between each repetition was assessed at 2 min while recovery between sets was 3 min.

#### 2.2.2. C Group

In the first weekly session, participants completed 3 sets of 2 sprints per 30 m. Participants rested for 2 min between each repetition, and recovery between sets was 3 min. In the second weekly session, participants completed 3 sets of 2 sprints per 15 m. Customarily, participants rested for 2 min between each repetition, while recovery between sets was 3 min.

### 2.3. Statistical Analysis

The averages of each subject for the different groups were calculated. Winsorization was applied to deal with outliers, replacing them with 5th and 95th percentile values. This technique allowed for greater robustness and reliability of the data. A Shapiro–Wilk test was performed to check the normality of the distribution of each parameter.

For normally distributed parameters, a paired *t*-test (Pre_Vs_Post) was applied to compare the PRE and POST results of the two groups. Additionally, a repeated measure ANOVA was conducted to assess the interaction effect between time (pre-post) and group (control vs. intervention), enabling a comprehensive comparison of the two groups at both treatment times. For statistically significant results, Cohen’s d was calculated to determine the effect (effect size). For the non-normally distributed parameters, a Wilcoxon signed-rank test (Pre vs. Post) was applied to compare the PRE and POST results of the two groups, and a Mann–Whitney U-test (E vs. C) was applied to compare the two groups at the PRE and POST treatment moments. For statistically significant results, the rank-biserial correlation coefficient (r) was calculated to determine the effect size. For between-group comparisons, Welch’s *t*-test was utilized due to its robustness against heterogeneity of variances.

## 3. Results

### 3.1. The Anthropometric Characteristics

The anthropometric characteristics of the participants are summarized in Table 1.

### 3.2. The Force Platform Data

The repeated measure ANOVA was conducted to assess the jump height (cm), CoM jump height (cm), take-off velocity (m/s), and maximum concentric power (kW) during the CMJ, with a specific focus on evaluating the efficacy of sled-integrated resisted sprint training by examining the interaction effect between time (pre-post) and group (control vs. experimental). This analysis allowed us to determine whether changes in performance metrics differed significantly between the two groups over time, thereby providing insights into the effectiveness of the training intervention. The analysis revealed a significant time × group interaction for the jump height, F(1, 17) = 16.45, *p* < 0.001, η^2^ = 0.492, indicating that the changes in jump height over time differed significantly between the control and experimental groups. Similarly, a significant interaction effect was observed for the CoM jump height, F(1, 17) = 6.47, *p* < 0.05, η^2^ = 0.276, suggesting a differential improvement between groups for this variable. In contrast, no significant interaction effects were found for the take-off velocity, F(1, 17) = 0.01, *p* = 0.939, η^2^ = 0.000, or for the maximum concentric power, F(1, 17) = 0.42, *p* = 0.526, η^2^ = 0.024. These results indicate that the intervention did not produce statistically meaningful changes in these parameters compared to the control group. Regarding the main effect of the single sources, time (pre-post), or group (control vs. experimental), this was not found to be significant for all of the analyzed parameters. The lack of significant main effects underscores that the significant interaction is not simply a reflection of global trends or pre-existing differences but rather an effect unique to the intervention. Overall, the findings suggest that sled-integrated resisted sprint training effectively enhanced jump height and CoM jump height, while its impact on take-off velocity and maximum concentric power remains inconclusive.

To compare the post-intervention jump height between the experimental and control groups, an unpaired *t*-test was conducted. The results indicated a statistically significant difference in jump height between the groups (t (17) = 2.2, *p* = 0.044). On average, participants in the experimental group exhibited higher jump heights compared to those in the control group, with a mean difference of −4.36 cm (95% CI [−8.8, −0.13]).

No other significant differences in force platform data were detectable. However, it is worth mentioning that the post-intervention CMJ—take-off velocity and CMJ—maximum concentric power showed a positive trend in the E group, while, similarly to the CMJ height, the maximum concentric power in the C group diminished.

In Table 2, the relevant force platform data obtained from the CMJ test are illustrated.

### 3.3. The 30 m Sprint Times

**Figure 2:**
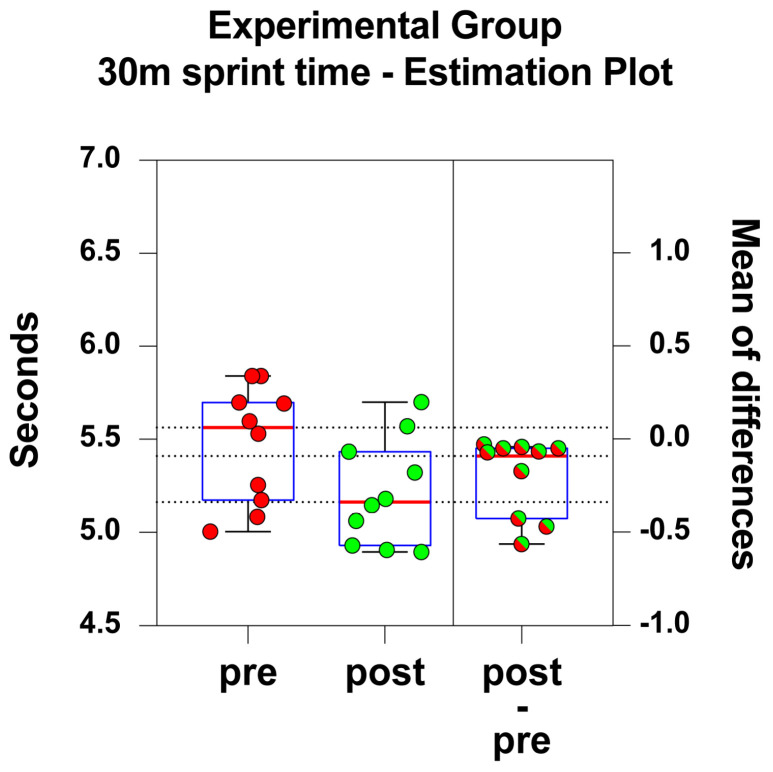
The E group 30 m sprint times are illustrated as scattered boxplots, where data are shown per subject. On the left side, red dots represent the pre-intervention data, pre; green dots represent the post-intervention data, post. On the right side, test differences are shown as green/red dots. Each box displays the median (red central line and black reference dotted line), 25th and 75th percentiles (blue box lower and upper edges), and the 5th and 95th percentiles (black lower and upper whiskers).

**Figure 3:**
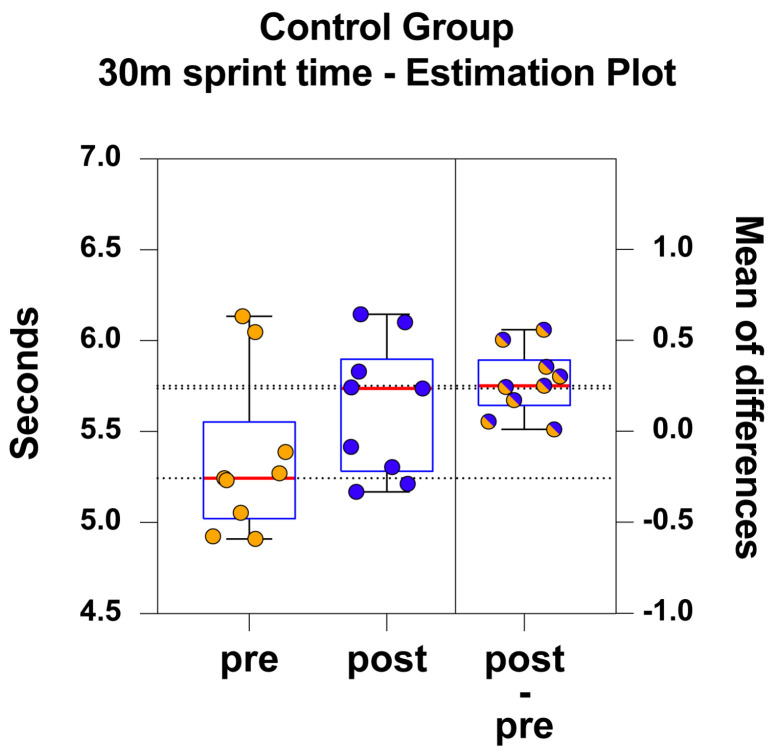
The C group 30 m sprint times are illustrated as scattered boxplots, where data are shown per subject. On the left side, orange dots represent the pre-intervention data, pre; blue dots represent the post-intervention data. On the right side, test differences are shown as blue/orange dots. Each box displays the median (red central line and black reference dotted line), 25th and 75th percentiles (blue box lower and upper edges), and the 5th and 95th percentiles (black lower and upper whiskers).

Estimation plots were used to illustrate the magnitude and the precision (i.e., the 95% confidence interval, CI) of the intervention effect for the E and C groups (Figure 2 and Figure 3, respectively). To assess the effect size between the groups, Cohen’s d was calculated. The value obtained for the comparison of the 30-metre sprint times between group E and group C was 1.253. According to Cohen’s guidelines, this Cohen’s d value indicates a very large size difference between the groups.

For the E group, the 30 m mean sprint time was 5.50 s (range ± SD = 5.00–6.13 ± 0.45) in the pre-intervention and 5.21 (range ± SD = 4.89–5.70 ± 0.28) in the post-intervention tests. The E group became (*p* < 0.01; t = 3.2; df = 9; two-tailed paired *t*-test) significantly faster after the six-week period.

The mean of the test’s differences was −0.29 s (range ± SD = −0.07–−0.47 ± 0.29) with the corresponding precision of CI (95% CI: −0.49–−0.08). Figure 2 illustrates the estimation plot and the mean of differences for the E group.

For the C group, the 30 m mean sprint time was 5.40 s (range ± SD = 4.91–6.13 ± 0.45) in the pre-intervention and 5.60 (range ± SD = 5.17–6.14 ± 0.37) in the post-intervention tests. Therefore, the C group became (*p* < 0.001; t = 4.4; df = 8; two-tailed paired *t*-test) highly significantly slower after the six-week period.

The mean of test differences was 0.27 s (range ± SD = 0.25–0.01 ± 0.18) with the corresponding precision of CI (95% CI: 0.13–0.41). Figure 3 illustrates the estimation plot and the mean of differences for the C group.

### 3.4. The aPHV Estimates

We calculated the estimated aPHV in our 13- to 14-year-old subjects two times, i.e., before and after the interventions, to check for possible variations induced by the experimental manipulations. The data are summarized in Table 3.

## 4. Discussion

Herein, we developed and tested a novel training program aimed at understanding whether and how sled-RST using both high and low resistive loads, set at different distances, can improve sprint and vertical jump performances in under-14-years-old football players.

The aim of this study was to understand if and how sled sprint training, through an integrated approach involving alternate sessions with high and low resistive loads, could improve sprint and vertical jump performances, which are fundamental athletic elements in soccer. Our hypothesis was that, through this integrated approach, athletes can reduce their sprint times while also increasing their force production.

We found in this study that sled sprint training integrating high (>20% BM) and low (≤20% BM) loads over different distances influences sprint and vertical jump performances in young male athletes aged 13–14 years.

The analysis of the 30 m sprint showed that the E group subjects became faster than they were before the six-week intervention (*p* < 0.01). This is not the case for the C group subjects who, after the six-week period, became even slower than before (*p* < 0.001). Our results demonstrate, then, that the E group is faster in the 30 m sprint compared to the C group. This confirms our starting hypothesis that sled-RST performed with higher loads improves sprint performance (cfr. Figure 2 and Figure 3).

In particular, the decrease in time necessary for E group subjects to complete the 30 m sprint can be attributed to at least two main reasons. It has been shown that light loads physiologically favor a reduction in contractile velocity by promoting the production of sprint-specific force [8]. This could allow for greater sprint power, seemingly resulting in a reduction in the time taken to perform the 30 m sprint. On the other hand, heavy loads (up to 115% of BM) could positively influence the pushing capacity to generate horizontal force through the effective application of force to the ground, as demonstrated by previous investigations [11,13,15]. Windwood et al. demonstrated the acute effects of strengthening using a sled with 75% body mass, which resulted in a significantly faster 15 m sprint at 12 min after the pull. Zisi M. et al. proved that heavy sled towing was an effective post-activation enhancement stimulus to improve sprint acceleration performance without impairing running technique. Results obtained by Cahill MJ. et al. [15] indicated that, in young athletes, heavier loads on the sled led to greater gains in short-distance sprint performance, especially in acceleration, than lighter loads. Also, the combined use of light and heavy loads has been already proven to be effective in different scenarios where athletes have improved their maximum dynamic and isometric resistance in rapid force development and power adaptations [16].

Additional information can be inferred by the increase in the 30 m sprint times found in the C group after the intervention. The increased 30 m sprint times observed in the C group most likely indicate that heavy sprint training can be regarded not only as a requisite to improve short sprints but could be, as we found in our experimental design, also significant to keep performance consistent over time, as already pointed out in previous experiences [9,31].

The platform data on the CMJ suggest a similar trend as that of sprint times for the E group, despite no significant differences in pre- and post-intervention measures of CMJ—take-off velocity and CMJ—maximum concentric power and in the CoM height being observed. Conversely, decreases in performance were visible in the CoM height of the C group, as already noticed by Guerra Jr who demonstrated that acute plyometric and sled towing stimuli improve the jumping performance of male football players [32]. These results could be directly ascribed to the integrated sled-RST training approach failing to enhance jump capabilities. Therefore, as already recognized, to improve jump abilities, heavy sled-RST training should be used [10]. Nevertheless, several other factors could have influenced the results. The specific training composition, the duration of the intervention, and the individual characteristics of the athletes could have played a significant role in the observed results. Also, there may be variations in individual responses to training, suggesting the importance of tailor-made training approaches to the athletes’ requirements and abilities [33]. However, the C group worsened slightly in velocity and power jump parameters. Although the control group continued their regular training, the absence of targeted interventions (such as resisted sprint training) might have led to a detraining effect. Over time, a lack of specific strength and power training could reduce muscle power output, thereby decreasing vertical jump height [34]. This suggests that using an approach that integrates both heavy and light loads could be useful to preserve fundamental bio-motor skills, as expected during periodization and session design [35,36], supporting that overload training can contribute to maintaining jump ability [32,37,38]. It appears that some specific parameters of vertical jumps, such as CMJ—CoM height, CMJ—take-off velocity, and CMJ—maximum concentric power, possibly require a greater training volume in the heavy load training session to be improved using this sled training approach.

The analysis conducted on the aPHV showed no differences between estimation data before and after the six-week period for both the E and C groups. The six-week period seems to have only a small positive effect on reducing the time it takes for each subject to reach aPHV. Imaginably, through training athletes with sled sprint training integrating high and low loads over different distances months or years earlier than what we did in this research, a greater effect could be achieved in the change of aPHV estimation.

This study contrasts with some findings in the literature [39,40]. It is not completely appropriate to say that training with resistive loads exceeding 20% of BM necessarily induces negative modifications in sprint biomechanics, as more in-depth studies have reached contradictory conclusions [9,19,41]. Clearly, any young athlete may respond differently to heavy sled training, with variations in peak adaptation times and magnitudes. It is in fact important to consider personalized training programs and different post-training test windows to achieve inclusive interpretations of training-induced adaptations [30,39]. If we want athletes to be more explosive and capable of moving at higher speeds, they must be able to apply greater force to the ground [42]. Consequently, more force is needed for more acceleration [43,44]. If the force component is limited by not providing adequate loads (e.g., using only resistive loads that are too light on the sled), RST would be used both out of context and not up to its full potential. In addition to being ecologically improper, it is also inadequate to always train with the same resistive loads on distances that are not consistent with the set loads [17,18,21].

Our study has some limitations. These include the relatively low number of subjects studied and the exclusive evaluation of the CMJ instead of considering other jump performances such as Standing Long Jumps, to explore the connection between sprint and jump abilities in the same horizontal plane. Although horizontal jump abilities do not fully translate to the field like vertical ones, it would be legitimate to include broader evaluations. It would be also interesting to examine hybrid jump abilities and their correlation with sprint performances. Another possible limitation could reside in the choice of the study population, composed of young male soccer players with adaptability and optimal training windows based on individual development. It would also be necessary to understand how training windows vary after a group of pre-PHV athletes is precociously subjected to strength training. Finally, adopting individualized plans rather than team plans is suggested to maximize quick and effective results.

## 5. Conclusions

We can conclude that sled-RST is a valid training method for improving sprint capabilities. The approach used in this study and applied to the sled-RST methodology will be called “sled-IRST” (integrated resisted sprint training). By employing both high and light loads over distances relative to the load, there is a reduction in sprint times over 30 m and an increase in vertical jump height. We encourage the use of strength training methodologies at the pre-PHV age of athletes without working specifically on hypertrophy.

Therefore, we encourage continued investigation into this topic to inform the process of developing evidence-based recommendations for scholars and trainers interested in enhancing sprint performance. There are several areas where future research is warranted, including longitudinal effects. Future studies should investigate the long-term effects of sled-IRST on athletic performance, particularly how sustained training over several months or even years impacts overall development and injury prevention in youth athletes. Neuromuscular adaptations should be researched and further studies are needed to explore the neuromuscular adaptations resulting from sled-IRST, particularly how varying load intensities influence muscle activation patterns, force production, and sprint biomechanics.

## Figures and Tables

**Table 1:** The anthropometric characteristics *.

Group	Status	Body Mass	Height	Sitting Height
		Mean ± s.d.	Mean ± s.d.	Mean ± s.d.
E (*n* = 10)	Pre	48.80 ± 10.50	1.63 ± 0.79	79.50 ± 2.65
E	Post	49.00 ± 9.10	1.63 ± 0.84	81.20 ± 3.16
C (*n* = 9)	Pre	51.11 ± 8.88	1.60 ± 0.11	82.60 ± 6.12
C	Post	51.11 ± 6.82	1.61 ± 0.14	83.50 ± 6.63

* No statistically significant difference across the groups and status (all *p* > 0.05).

**Table 2:** Force platform data from the CMJ test.

Group	Status	CMJ—CoM Height (cm)	CMJ—Jump Height (cm)	CMJ—Take Off Velocity (m/s)	CMJ—Maximum Concentric Power (kW)
		Mean ± s.d.	Mean ± s.d.	Mean ± s.d.	Mean ± s.d.
E (*n* = 10)	Pre	33.04 ± 5.92	22.15 ± 4.84	2.27 ± 0.23	1.99 ± 1.51
E	Post	33.59 ± 4.23	22.47± 4.68	2.24 ± 0.36	2.01 ± 0.62
C (*n* = 9)	Pre	31.54 ± 5.92	20.90 ± 4.40	2.24 ± 0.37	2.11 ± 0.51
C	Post	29.39 ± 5.00	19.57 ± 4.07	2.23 ± 0.38	1.99 ± 0.43

**Table 3:** The estimated aPHV before and after intervention *.

Group	Status	Predicted aPHV
		Mean ± s.d.
E	Pre	14.2 ± 0.38
E	Post	14.1 ± 0.50
C	Pre	13.8 ± 0.89
C	Post	13.8 ± 0.91

* Two month intervals. No significant differences were detectable for the estimates of aPHV either between groups or in both groups between pre- and post-intervention periods (all *p* > 0.05).

## Data Availability

The data presented in this study are available on request from the corresponding author.
